# Effects of Soundscape Complexity on Urban Noise Annoyance Ratings: A Large-Scale Online Listening Experiment

**DOI:** 10.3390/ijerph192214872

**Published:** 2022-11-11

**Authors:** Andrew Mitchell, Mercede Erfanian, Christopher Soelistyo, Tin Oberman, Jian Kang, Robert Aldridge, Jing-Hao Xue, Francesco Aletta

**Affiliations:** 1 Institute for Environmental Design and Engineering, The Bartlett, University College London, London WC1H 0NN, UK; 2 Institute for the Physics of Living Systems, University College London, London WC1E 6BT, UK; 3 Institute of Health Informatics, University College London, London NW1 2DA, UK; 4 Department of Statistical Science, University College London, London W1T 7PJ, UK

**Keywords:** sound source recognition, noise annoyance, sound perception, soundscape, urban environments

## Abstract

Noise annoyance has been often reported as one of the main adverse effects of noise exposure on human health, and there is consensus that it relates to several factors going beyond the mere energy content of the signal. Research has historically focused on a limited set of sound sources (e.g., transport and industrial noise); only more recently is attention being given to more holistic aspects of urban acoustic environments and the role they play in the noise annoyance perceptual construct. This is the main approach promoted in soundscape studies, looking at both wanted and unwanted sounds. In this study, three specific aspects were investigated, namely: (1) the effect of different sound sources combinations, (2) the number of sound sources present in the soundscape, and (3) the presence of individual sound source, on noise annoyance perception. For this purpose, a large-scale online experiment was carried out with 1.2k+ participants, using 2.8k+ audio recordings of complex urban acoustic environments to investigate how they would influence the perceived noise annoyance. Results showed that: (1) the combinations of different sound sources were not important, compared, instead, to the number of sound sources identified in the soundscape recording (regardless of sound sources type); (2) the annoyance ratings expressed a minimum when any two clearly distinguishable sound sources were present in a given urban soundscape; and (3) the presence (either in isolation or combination) of traffic-related sound sources increases noise annoyance, while the presence (either in isolation or combination) of nature-related sound sources decreases noise annoyance.

## 1. Introduction

The World Health Organization (WHO) reports environmental noise as the second most important cause of ill health in Europe, behind only air pollution [1]. It is a major public health issue that can lead to negative cardiovascular and metabolic effects, reduced cognitive performance in children and compromised mental health [2], as well as severe annoyance and sleep disturbance [3]. Long-term exposure to environmental noise is estimated to cause 12k+ premature deaths and to contribute to 48k+ new cases of ischemic heart disease per year, in Europe alone [4]. The WHO estimates that 22 M people suffer chronic high annoyance, which is associated with specific outdoor sources of environmental noise [3]. Exposure-response curves are indeed available in literature to estimate such effects (e.g., aircraft noise eliciting proportionally more annoyance than road traffic noise, which is in turn more annoying than railway noise, etc.) [5].

Noise annoyance is often considered as the main effect of noise and understood as a “multi-faceted psychological concept, including behavioural, and evaluative aspects” [6]. Indeed, it is generally acknowledged that while noise annoyance partly relates to acoustic variables, the acoustic characteristics of the exposure do not necessarily play a major role in the formation of this perceptual construct. Therefore, scholars have started looking at different acoustic- and listeners-related factors, such as spectral features [7,8], the visibility of the sound source [9,10], interactions with other perceptual modalities (such as, visual perception [11], smell perception [12]), personality traits [13], psychological well-being [14] and so on.

The ISO/TS 15666:2021 [15] defines annoyance (specifically “noise-induced annoyance”) as “one person’s individual reaction to noise.” Such construct is analysed through socio-acoustic surveys based on questions with either verbal or numerical rating scales. Within the ISO/TS 15666 context, the noise annoyance scales refer to long-term exposures, typically in residential settings and for a specific sound source (i.e., “Thinking about the last (12 months or so) …”). On the other hand, soundscape studies also make use of the concept of annoyance/annoying when referring to individuals’ experience of a stimulus and tend to focus on more short-term and contextual reactions to a given acoustic environment (see, e.g., [16,17]).

Previous research has looked at models that predict the annoyance caused by combinations of noise sources, yet the sources of interest in such cases still refer to conventional transportation noise (i.e., aircraft, road traffic, or railways) (e.g., [18]). Only more recently have researchers started looking at a broader range of sound sources that are more likely to be experienced in everyday life, how similar acoustic environments combinations are to one another, and how these aspects contribute to noise annoyance formation (e.g., [19,20,21]). Along with developments in the definition of environmental sound source taxonomies [22,23,24], these studies have demonstrated the impact of the semantic meaning of sound sources on the affective perception of the soundscape. However, they have not yet explored how different combinations of sound sources (e.g., bird song AND water sounds AND traffic noise) can explain the perceptual impact differently than considering the sources individually.

In conclusion, there is general consensus now on the fact that approaching noise annoyance as a one-dimensional problem in the urban realm—e.g., monitoring the “exposure” in terms of noise levels, or focusing on the semantics of a limited set of sound sources, is not sufficient, and there is a need to look at the perception of environmental sounds from different perspectives (e.g., with bottom-up processing, stimulus-driven and source-oriented approaches) that will eventually improve how we manage soundscapes in cities [25,26], which is very much in line with the concept of soundscape studies that especially looks at changes between wanted and unwanted sound sources in different contexts [16,27,28].

Therefore, the aims of this paper are (a) investigating whether different combinations of sound sources in complex urban acoustic environments may result in different outcomes in terms of noise annoyance (Research Question #1); (b) investigating whether the number of perceived sound sources, which in this study is defined as “soundscape complexity”, influences noise annoyance (Research Question #2); (c) investigating whether there is an association between the presence of specific sound sources in an urban acoustic environment irrespective of the overall combination and an increase/decrease in noise annoyance (e.g., how the presence of bird tweets may increase/decrease the annoyance, regardless of the other sources present) (Research Question #3).

For this purpose, a large-scale online experiment was conducted using audio recordings from a large international database of complex urban soundscapes [29]. The main benefit of this database is that it represents realistic urban acoustic environments, as opposed to a large corpus of literature on noise annoyance that uses simpler signals/stimuli, hence potentially resulting in less ecologically valid results.

## 2. Materials and Methods

### 2.1. Platform and Participants

A remote active listening experiment was designed and hosted on Gorilla Experiment Builder (www.gorilla.sc), a professional online platform used for studying complex behaviours [30]. The survey was distributed via Prolific (www.prolific.co) to a pool of pre-registered participants, and data collected between 5 July 2021 and 23 July 2021 (the online data collection tool was kept active for as long as necessary to reach the target number of participants).

The study was first advertised in Prolific. Before the main (active) listening experiment on Gorilla, registered participants went through pre-experiment screening steps in which the researchers made sure that they met all the inclusion criteria of the study. The pre-screen steps were carried out in Prolific as soon as the participants signed up for the study. The participants were informed of the requirements including wearing headphones and sitting in a quiet room. They were provided with a brief description about the objectives of the study, what they were expected to do during the active listening experiment, how much they would be paid on successful completion of the study, and system requirements (e.g., using google Chrome, and desktop computer). It was clarified to them that if they failed to meet the system requirements, they did not successfully finish the active listening experiment or they did not achieve a performance level above 70%, they would not be compensated. The main goal of the online experiment was collecting data from a relatively large pool of participants being exposed to a given set of complex urban acoustic environments (i.e., multi-source), on noise annoyance level and sound sources recognition. Furthermore, via pre-experiment screening, data collection aimed at individuals representing certain demographics, i.e., 18- to 60-year-old, gender-balanced sample. The age range was decided to ensure hearing loss due to ageing was less likely to be present in the sample, and in terms of hearing ability, the participants would be sufficiently homogenous [31]. Participants reported an absence of otologic or cognitive disorders, history of epilepsy, or index neurological event. It is noteworthy to mention that the advertised study was not visible to the participants who did not meet the inclusion criteria (e.g., age and gender), which was provided by the participants when they had first registered in Prolific. Once the participants passed the pre-experiment screening (e.g., healthy hearing and using headphones), they clicked on the provided link that directed them to the online active listening experiment hosted by Gorilla.

With the growing need of developing reliable protocols for online listening experiments, especially considering the constraints imposed on the laboratory settings for researchers around the world during the COVID-19 pandemic, the methodological approach of the two platforms mentioned above is now generally accepted in the environmental noise academic community, provided that a few quality checks (specified below) are put in place [32].

### 2.2. Auditory Stimuli

The dataset of stimuli (audio recordings) included 2890 total recordings, sourced from the International Soundscape Database v0.5 (ISD) [29,33], which is a large dataset of urban acoustic environments recorded in different regions of the world. The original ISD recordings were collected along with in situ soundscape surveys to characterise the soundscapes of urban spaces in London and Venice, according to the SSID Protocol [33]. These data were collected by recruiting users of the public space to be assessed and asking them to complete a soundscape survey. While participants were completing this survey, a binaural recording of approximately 30 s was made to capture the exact sound environment to which the participant was exposed. In total, 13 public spaces were assessed (including surveys) throughout 2019 and additional recordings were collected in the same locations during the 2020 COVID-19 lockdowns (not including the participant surveys) [34]. In total, this dataset includes 2519 recordings with a duration between 8 and 122 s. For the purposes of this study, only the calibrated binaural recordings are used from the ISD, and not any additional information about the in situ participants, locations, or soundscape assessments.

**Figure 1:**
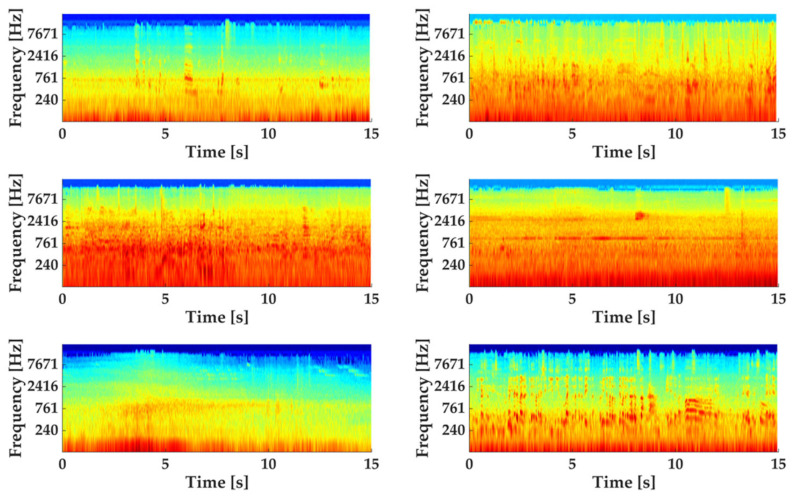
Log spectrograms of six examples from the set of stimuli used in the experiment.

In order to standardise the length of recordings used in this study and expand the number of available recordings, the original recordings were split into 15-s chunks, resulting in 2890 recordings. This operation was performed using the pydub package (v0.25.1) in Python [35] where the full WAV file is loaded in, then 15-s chunks are extracted starting from the beginning of the recording (i.e., a 47-s-long original recording would result in three 15-s recordings, with the remainder 2 s discarded) and exported as MP3 files to be uploaded to the online survey platform. Some examples of Log spectrograms for the stimuli used in the experiment are shown in Figure 1. While it was not possible to control for the absolute sound pressure levels presented to participants in the online listening task, the relative loudness between the sound sources present across the pool of audio samples can be considered faithful to a typical acoustic environment experienced on-site.

### 2.3. Experimental Procedure

**Figure 2:**
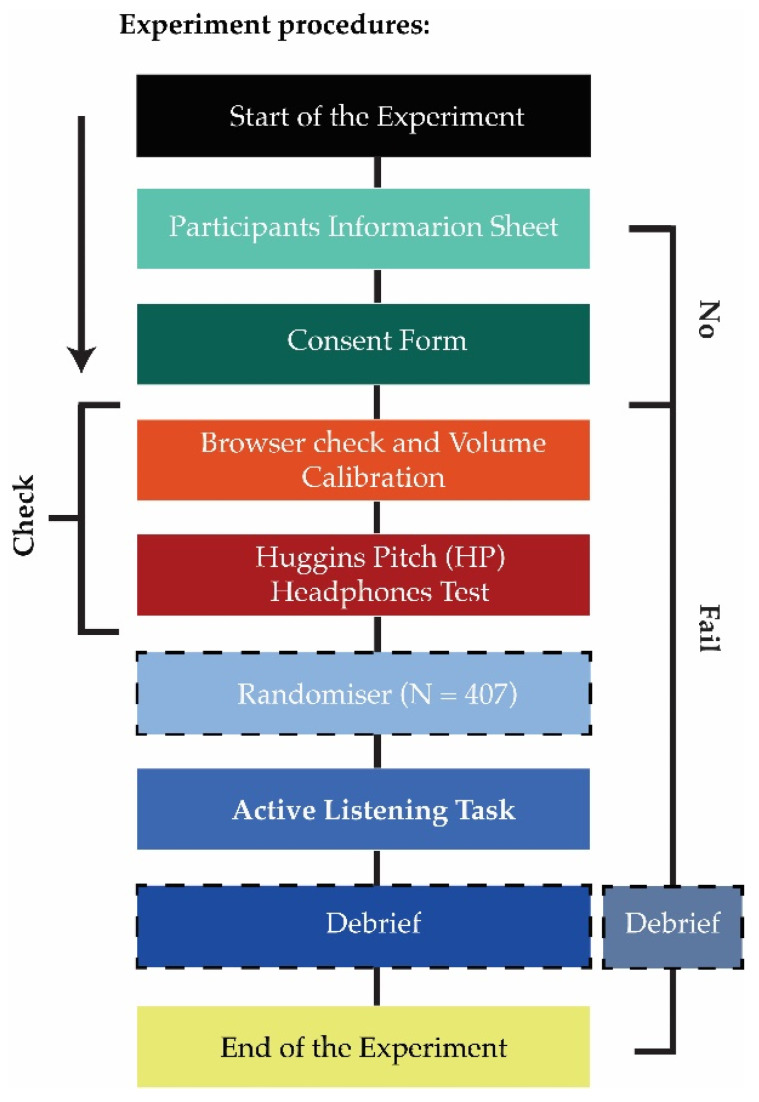
Progression of the online experiment in the Gorilla platform: only participants providing consent and passing both browser sound and headphone checks (highlighted with the black vertical line labelled “Check” on left) would begin the active listening experiment. If the participants did not read the information sheet, would not consent, or failed the browser, volume calibration or Huggins Pitch (HP) headphones test, they were debriefed and directed to the end of the experiment (highlighted with the black vertical line on the right).

Since the accuracy of sound recognition and noise annoyance rating was the core target of the experiment, every effort was made so that participants qualified for the study and their equipment could deliver the sound stimuli properly. Hence, the online experiment was designed to minimize possible study biases and environmental confounders (Figure 2).

#### 2.3.1. Browser Sound Check and Volume Calibration

Prior to the main tasks, the participants first read the information sheet and consent to participate in the study. Then, they were instructed to adjust their browser setting and playback volume in a few steps. The first step was done by playing five-second music sample, so the participants could confirm if their browser supports audio playback and if they heard the music piece, and if not, there were directed to another page, instructing them to change the browser configurations. If this browser check had failed, it would have led to rejection. Following the browser check, the participants were led to the volume calibration in which they were asked to set the volume of their headphones as high a level of their comfortable loudness level by increasing the volume from zero while a white noise sample was played back, previously matched at −11.06 dBFS (total Root Mean Square), corresponding to loud stimuli from the dataset. On the same page, the participants were asked to listen to a typical quiet audio sample from the dataset (−28.8 dBFS, total RMS) so to ensure they will be able to perceive all the samples. After confirming that they can ‘hear the sound clearly through their headphones’, they were strictly asked not to adjust their listening setup any further and were allowed to proceed to the next task.

#### 2.3.2. Huggins Pitch Test (HP)

Once the browser settings were compatible for running the experiment and the volume was set to ensure participants’ comfort and sufficiently accurate playback levels, the participants proceeded to the headphones check test, the dichotic Huggins Pitch test (HP), a validated test for online studies [36]. The HP consisted of one block, including six trials. Each trial includes three intervals of white noise, each 1-s long. The third interval contained the HP stimulus. A centre frequency of 600 Hz was used, and the interval order varied randomly across the block and the participants. Once the participants passed the HP test, they proceeded to the main experiment. One thousand seven-hundred and twenty-three individuals were initially recruited. Since not all headphones can deliver the quality of sound and frequency range, N = 502 participants were excluded automatically from the study, including N = 485 participants who were rejected due to time limit (60 min) and N = 17 participants who withdrew from the experiment voluntarily.

#### 2.3.3. Active Listening Tasks

**Figure 3:**
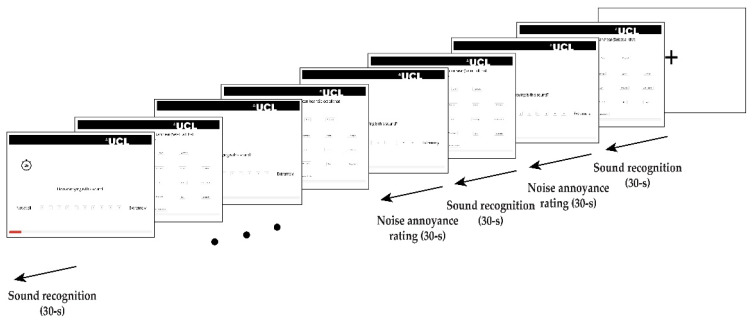
The online paradigm: A standard block design was used which auditory stimuli were presented for 15 s, followed by 15 s for sound sources labelling; after the sound source recognition, the same stimulus was presented again for 15 s and followed by 15 s for the annoyance rating task.

A total of N = 1221 participants successfully completed the experiment (M_age_ = 30.6, SD_age_ = 10.2; Ethnicity: Asian 9.5%, Black 4.5%, Mixed 5.9%, Other 2.0%, White 78.1%—Sex: F 45.7%, M 54.3%; demographic questions were optional, so data is reported for participants who provided the information). The main experiment consisted of two tasks: sound recognition and annoyance rating initiated with the instruction and a practice block comprising five trials similar to the main experiment. During the active listening experiment, participants listened to ten 15-s-long binaural recordings of urban environments and were instructed to select all the sound sources they could identify within the recording by asking “*Please choose the sounds you can hear (select all that apply)*”. For the sound source recognition task, participants were provided with a list of 24 labels they could select from. This included 22 sources from a subset of sounds provided in the sound source taxonomy used by Salamon, Jacoby, and Bello [22]. The included sources were chosen from the taxonomy by comparing them to a preliminary source identification performed by the researchers while cleaning/preparing the recordings. Those sounds that had been identified in this preliminary round, as well as others which were considered likely to appear, were selected. The sound source labels included dog bark, screeching brakes, general traffic, rail, bird tweet, aircraft, footsteps, music, rustling leaves, siren, car, construction, motorcycle, ventilation, shouting, speech, horn, laughter, children, bells, bus, and water. In addition, a “non-identifiable” option was added for those sources which listeners could identify as a single source but was unsure what it was (selected for 8.7% of recordings), and an option “other” for any sources which listeners could identify but which had not been provided in the list (selected for 13.5% of recordings). The layout of the 24 sound source labels varied randomly across the participants. For each recording, they had 30 s in total, including 15 s for sounds presentation/playback and 15 s for sound sources selection. For the annoyance rating task, participants were instructed to assess the noise annoyance they perceived for that stimulus on a discrete 10-point scale, ranging from 1 (not at all) to 10 (extremely) by asking “*How annoying is this sound?*” (see: Figure 3). It should be noted that the definitions of “noise annoyance” may vary depending on context, discipline, applications, etc. Since the attribute used in this online experiment was “annoying” and because of the focus on sound, the variable will be referred here as “noise annoyance” or “annoyance”. For the sake of analysis and conclusions to be drawn, it should rather be interpreted as “relative annoyance”. For each participant, ten recordings were randomly presented out of the pool of 2890 excerpts, and the recordings were selected such that each recording was presented at least to more than one participant. The experiment took approximately 15 min to complete, and the participants received a small compensation for their time (£1.88).

The recording stimuli used in the listening task and the response data are openly available on Zenodo (https://doi.org/10.5281/zenodo.7158057) [37].

## 3. Data Analysis

In the original data, each recording was assessed by between two and four participants. Since each participant was answering independently, each recording is associated with several sets of recognised sound sources (either individual sources or combinations), as well as several annoyance scores. To collapse these into a single set of sound sources per recording, a “majority” approach was considered, i.e., if most participants assessing a given recording identified a source as being present in it, this source was considered to be effectively present. This resulted in a 2890 by 24 data frame (2890 recordings, each with up to 24 possible labels present). On average, each recording has 3.1 identified sound sources present. Regarding the annoyance scores, they were averaged within the group of participants assessing any given recording, to get a unique annoyance score for each recording.

## 4. Results

To address the three research questions of this study, the analysis of the results was carried out on different “layers”. Firstly, the dataset was approached as a whole and subjected to association rule learning and clustering algorithms to investigate the effect of sources profiles and combinations. As a separate step, the recordings were sorted according to the number of sound sources that participants had identified in them. This could be seen as a proxy for what in this study we consider to be the perceived “soundscape complexity” of the acoustic environment, and noise annoyance as a function of this variable was then assessed accordingly. Finally, recordings were stratified as per whether they contained a specific sound source (i.e., one of the 24 possible labels), either alone or as part of a set of sources.

### 4.1. Noise Annoyance as a Function of Sound Sources Combinations (Research Question #1)

The first step of analysis consisted in clustering the dataset of responses for the identified sound sources for the sake of detecting groups of acoustic environments with similar profiles. The goal of this clustering analysis was to identify patterns in how sound sources appear together within an acoustic environment. Each recording was characterised by a set of 24 binary values indicating whether a source was present (1) or not (1). The Jaccard distance [38] was used to characterise the similarity or diversity of these sets for each recording compared to every other recording, resulting in a 2890 by 2890 dissimilarity matrix. Hierarchical agglomerative clustering analysis was then used. However, this path of investigation did not lead to any meaningful result, as the different sound sources profiles emerging from the cluster analysis did not result in statistically significant differences in terms of noise annoyance (i.e., annoyance scores did not vary significantly as a function of cluster membership). In addition, upon examining the clustering results, the researchers were unable to determine any useful conceptual model for describing the identified combinations of sound sources.

**Figure 4:**
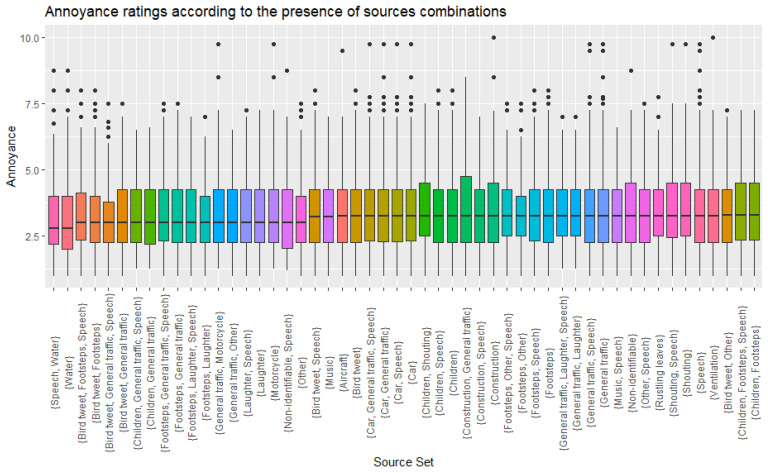
Box plots of the Annoyance scores for the recordings as a function of sound source present (including the “other” and “non-identifiable” labels) and sources likely to be present together in the same recording as per the association rule algorithm.

Subsequently, instead of exploring all possible combinations of sound sources (which would be impractical), an approach based on “association rule learning” was used; this is a machine learning method derived from the marketing and product fields for analysing how products tend to appear together in purchase datasets [39]. In the context of this study this was aimed at identifying sets of sounds sources that are more likely to appear together in any labelled recording. However, when looking at the effect of being exposed to acoustic environments with specific sound sources recurring together on noise annoyance, no meaningful differences could be observed. This can be clearly seen in Figure 4, where the distributions of noise annoyance scores are plotted for different groups of recordings with recurring sound sources identified in them. The negative results of these approaches indicated that, contrary to one of the initial primary hypotheses, the particular combination of sound sources which co-occur are not useful for determining annoyance scores for a complex soundscape.

### 4.2. Noise Annoyance as a Function of Soundscape Complexity (Research Question #2)

**Figure 5:**
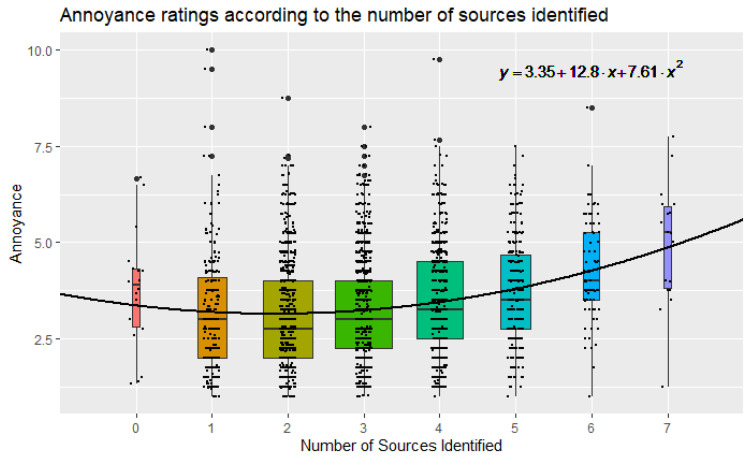
Box plots of the Annoyance scores as a function of the number of sound sources present in the recording (identified by participants), box width represents relative sample size; the N = 8 sound sources group only included two datapoints, and was therefore dismissed (the mean annoyance scores for those is M = 5.0).

Since the range of number of identified sources (i.e., labels assigned by participants) per recording varied between 0 and 8 in the dataset, the sample was organised into a set of nine mutually exclusive sub-sets with increasing soundscape complexity. When plotting distributions of annoyance scores according to soundscape complexity, as shown in Figure 5, an interesting pattern emerged: mean noise annoyance was minimized for N = 2 sound sources identified, with scores increasing when soundscape complexity was heading towards either N = 0 or N = 8 sound sources identified (however, the N = 8 sound sources group only included two datapoints, and was therefore removed from Figure 5 for clarity; the mean annoyance scores for those is M = 5.0). A one-way ANOVA was then conducted to determine if the noise annoyance was significantly affected by the soundscape complexity dimension. The Annoyance score was statistically significantly different between different groups, *F*(8, 2881) = 15.354, *p* < 0.001. Post hoc tests (Bonferroni) revealed that such outcome was mostly driven by differences between the N = 2 sources group and the N = 4, 5, 6, 7 sources groups, which can be observed again in Figure 5. A second-degree polynomial regression was fit to indicate the relationship between the number of sound sources (i.e., soundscape complexity) to the reported annoyance scores, and is shown in Figure 5.

### 4.3. Noise Annoyance as a Function of Individual Sound SOURCES presence (Research Question #3)

**Figure 6:**
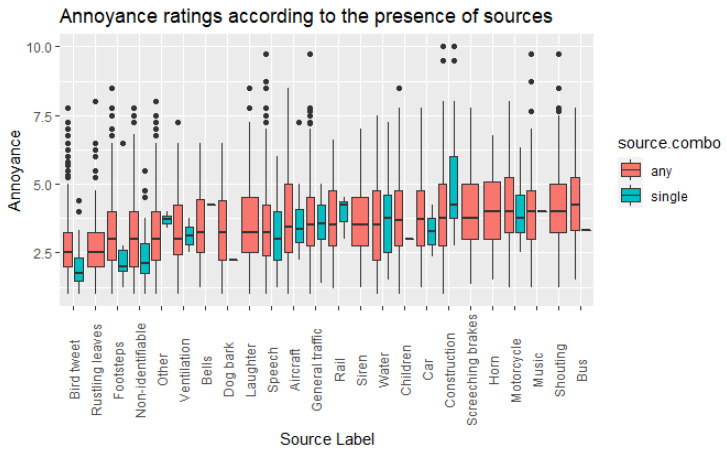
Box plots of the Annoyance scores as a function of sound source presence, either on its own (“single”) or in combination with other sources (“any”), including the “other” and “non-identifiable” labels; box width represents relative sample size.

As an additional layer of analysis, it was decided to look at the presence of specific sound sources as a possible factor of influence on the perceived noise annoyance assessed by participants. Distributions of mean annoyance scores as a function of whether a source was identified in a recording, either on its own or in combination with any other source, were plotted, as shown in Figure 6. It can be clearly seen that there is some variety across sources, with the presence of traffic-related sources being generally associated with higher annoyance scores, as opposed to nature-related and backgrounded sound sources, where annoyance is lower.

To confirm this pattern, a set of independent-samples *t*-tests was run for each of the 24 labels (sound sources) of the active listening experiment to determine if there were differences in annoyance scores between the conditions when the sound source was present (either on its own or in combination with other sources) in the recording (“Yes”), and when it was not (“No”). An alternative Welch *t*-test was considered whenever the assumption of homogeneity of variances was violated.

Table 1 shows that basically most comparisons resulted to be different in a statistically significant way, except for the labels Bells, Dog bark, Water, Ventilation. For the sound sources: Aircraft, Bus, Car, Children, Construction, General traffic, Horn, Laughter, Motorcycle, Music, Rail, Screeching breaks, Shouting, Siren, Speech, and Water, the noise annoyance increased when the source was present, compared to when it was not. Conversely, for the sound sources: Bird tweet, Footsteps, Non-identifiable, Other, and Rustling leaves, the noise annoyance scores decreased when the source was present compared to when it was not.

## 5. Discussion

Although past studies have advanced our understanding of the effect of distinct sound sources on noise annoyance perception, to the best of our knowledge, the results were hampered by some limitations. Previous studies have either focused on conventional noises such as road traffic noise or single-sourced noises like aircraft noise [40,41], hindering the ecological validity of the sounds and sound sources combinations in the context of urban areas. To address this limitation, we utilized a large sound dataset, recorded from several locations with multiple source noises such as cars, construction, rail and so on. In addition, existing studies applied small sample sizes or focused participant groups like students [42], whereas in the current study we recruited many participants representing both sex, different age groups, and ethnicity. Most studies to this date have drawn conclusions based on the sounds that were influenced by undesirable environmental stimuli such as visual stimuli [43]. Given that other sensory modalities (e.g., visual stimuli) may confound the noise annoyance perception [10,44], we isolated the sound stimuli to minimize the effects of other modalities by adopting a controlled online active listening experiment.

### 5.1. Effect of Sound Sources Combinations (Research Question #1)

Previous studies have advanced our understanding of the effect of specific sound sources on noise annoyance perception [45,46,47,48]. One of the hypotheses of this study was that combinations of different sound sources would affect differently the noise annoyance scores returned by participants. The thought was that, for instance, while traffic noise is known to be annoying, this effect would change depending on the other sources present. Likewise, that although bird sounds decrease annoyance, perhaps they are most effective when paired with another positive sound such as footsteps. However, our results have indicated this is not the case, and the positive or negative semantic effect of a sound is most important on its own, independent of the other sources present. This was observed despite testing different associations strategies for the sound sources profiles. Hence, the focus was shifted towards other potentially important aspects of the complex urban acoustic environments.

### 5.2. Effect of Number of Sound Sources, or “Soundscape Complexity” (Research Question #2)

Another main goal of this investigation was testing whether the number of identified sound sources in an urban acoustic environment may play a role in the noise annoyance assessment by people, which was broadly defined as soundscape complexity. The concept of “complexity” is not new in soundscape studies, but it has so far been used mostly in acoustic ecology and ecoacoustic applications [49]; here, it is interpreted from a human listener’s perspective. When listening to a soundscape, the listeners may perceive a single dominant sound source, they may perceive multiple overlapping, coincident, or related sound sources, or they may be unable to perceive any identifiable sounds at all. An interesting pattern emerged, showing a relatively high average noise annoyance score with 0 sources identified, dropping for 1 and 2 sources identified, to increase again towards 8 sound sources identified. This seems to suggest that fewer clearly distinguishable sound sources in a given soundscape may be preferrable (and less annoying) than either no sources (i.e., monotonous/uneventful soundscape) or too many sources (i.e., chaotic soundscape). This finding seems to be in line with Schafer’s theory of hi-fi soundscapes being more pleasant than lo-fi, blurred soundscapes [50]; thus, hi-fi soundscapes would correspond to lower soundscape complexity and lo-fi soundscapes would correspond to higher soundscape complexity, either due to many overlapping identifiable sources, or so much noise that no single source is identifiable. Furthermore, this is also aligned with the information load theory for soundscape studies previously proposed by Axelsson. Information Load was hypothesized to be one of the main dimensions underlying soundscape experience and assessment [51], relating to the interaction between soundscape complexity and the individuals’ ability to comprehend and process information. The assumption is that a lower information load (i.e., lower soundscape complexity) will be perceived as an unpleasant/monotonous/uneventful soundscape; a moderate information load (e.g., a quiet natural soundscape) will be perceived as pleasant; a slightly above moderate information load (e.g., an eventful soundscape with some moderate violation of personal expectation, pleasantly surprising) will be perceived as exciting; while a very high information load (e.g., a chaotic soundscape, overwhelming to process) will be perceived as annoying.

Something else to consider in the context of the sound sources recognition task is that, despite having been given up to 24 labels to choose from, the maximum number of sources ever identified by participants in any given audio recording was eight. This is somehow an interesting empirical confirmation of the protocol of Method B of the ISO/TS 12913-2:2018, methods for data collection, where the protocol instructs participants of a soundwalk to *“…list sound sources you noticed in descending order, starting with the most noticeable sound source. Any number of listed sound sources is possible, but limited to 8*” [52]. To the best of our knowledge, the choice for a maximum of eight source was not justified in the ISO document, but our findings may support the hypothesis that 7–8 sources is the operational maximum of sources that participants can holistically pay attention to in a listening task.

### 5.3. Effect of Specific Sound Sources (Research Question #3)

Following the outputs of the previous steps of analysis, a more in-depth analysis of the presence/absence of single sound sources in the soundscape recordings also revealed further details. This was driven by the proposition that some specific sound sources would be more likely to be associated with higher annoyance. For instance, Gille and colleagues [53], in their survey in residential sites exposed to different sound sources and source combinations, observed that annoyance ratings for “total annoyance” were the highest any sound source combination that would include including aircraft noise, even when the sound level of the second source (e.g., road traffic) was higher that aircraft noise.

As expected, when sound sources related to road traffic (but also transport and industry more generally) were present, noise annoyance was significantly higher (e.g., Aircraft, Bus, Car, General traffic, Horn, Motorcycle, Rail, Screeching breaks, Siren); although the presence of some unexpected sources, like Children, Laughter, Music, or Water, was also found to be associated with increased annoyance. This reinforces the concept that sources should not be treated in isolation but as part of a complex sound environment. Looking at the associations between presence of sound sources and decreased noise annoyance instead, the relevant labels were: Bird tweet, Footsteps, Non-identifiable, Other, and Rustling leaves; hence, the types of sound sources that would likely be found in a quiet natural soundscape/context. Likewise, findings confirm the potential that pleasant natural sounds have in reducing stress and offering opportunities for restoration [54]. Interestingly, when a non-identifiable sound source was identified in the recordings, annoyance scores were lower. A possible explanation for this is that participants may have perceived these non-identifiable, backgrounded/distant sources as an indicator of some “urban buzz” and lively context—considering that data collection took place during the COVID-19 pandemic, when most people had had some experience of lockdown and restriction in social interactions, one could hypothesize that sources relating to human activity may have been interpreted as a much-desired “normalcy” [55,56,57]. Yet, it is worth noting that, considering the pool of labels used in the experiment, the ones associated with decreased annoyance represent a much smaller proportion of it. This suggests that acoustic environments should be carefully designed as the opportunities for adding positively perceived sounds to urban soundscapes are not abundant.

## 6. Conclusions

In this study, a large-scale online active listening experiment was carried out with 1.2k+ participants and 2.8k+ audio recordings of complex urban acoustic environments to investigate how the complexity of the soundscape—in terms of presence, number and combination of different sound sources would affect the perceived noise annoyance. The main conclusions of this study are:

- Combinations of sound sources in soundscape recordings is less important than the actual number of sound sources identified in the acoustic environment (which in this study we defined as “soundscape complexity”).
- A combination of any two clearly distinguishable sound sources (low soundscape complexity) in a given urban soundscape appears to minimize the perceived noise annoyance, which is higher instead, when the number of sources either increases or decreases.
- The presence of sound sources related to road traffic in a soundscape is associated with higher noise annoyance, while the presence of natural sound sources is associated with lower noise annoyance (even when road traffic noise is present).

For future studies, it will be important to focus attention on models for annoyance that consider sound sources with relatively higher “ecological frequency” [58]—i.e., that are more likely to be experienced by average persons during everyday life in urban contexts.

It is worth pointing out that scientific debate around the multi-faceted concept of noise annoyance, is quite broad and diverse. It should be acknowledged that in the context of this study the reader may notice some slight deviations from the framework provided by -inter alia- international standards for surveying noise annoyance (e.g., [15,59,60,61]). Of course, the formation of noise annoyance should be mostly looked at in context and in a holistic way, but that even within the limits of a semi-controlled experiment like the one conducted in this study (i.e., where the focus is on listening), building around the concept of “annoyance” would be most useful to explore some basic principles of sound sources combinations perception by users. The definition of noise annoyance provided by Guski et al. [6] appears to be broad/inclusive enough to cover such methodological approaches.

Many annoyance-focused laboratory studies have experimental designs similar to the ones adopted in this study and use wording to seek responses on annoyance ratings that sometimes are deliberately adapted from ISO/TS 15666 (which is broadly accepted, even if ISO/TS 15666 refers to long-term residential exposure) or phrase the question on “annoyance” in similar ways to the protocol in this study [62,63]. Different approaches—i.e., either the community noise and disturbance approach underlying the ISO/TS 15666:2021, or the soundscape approach underlying the ISO/TS 12913-2:2018—that deal with the perceptual construct of “annoyance” should be considered valid and useful. Indeed, the international community seems now to be putting effort to bridge the gap between these theoretical stances, which is also reflected in the ISO/TC 43/SC 1 (Noise) recently establishing its Working Group 68 to develop the ISO/AWI TS 16755-1 (Acoustics—Non-acoustic factors—Part 1: Definition and conceptual framework). This will hopefully lead to further research on the noise annoyance construct and a better understanding of this complex concept.

## Figures and Tables

**Table 1:** Descriptive statistics for the annoyance scores for each sound source and *t*-tests for each sound source between the groups when the source is present and when it is not.

			Annoyance Scores			*t*-Test for Equality of Means			
Source	Present	N	Mean	Std. Deviation	Std. Error Mean	*t*	df	Sig.	Mean Difference
								(2-Tailed)	
Aircraft	Yes	118	3.71	1.61	0.15	2.533	124.41	0.013	0.38
	No	2772	3.33	1.38	0.03				
Bells	Yes	54	3.44	1.20	0.16	0.489	2888.00	0.625 ^1^	0.09
	No	2836	3.35	1.40	0.03				
Bird tweet	Yes	932	2.71	1.16	0.04	−19.034	2168.24	0.000	−0.94
	No	1958	3.65	1.39	0.03				
Bus	Yes	83	4.31	1.42	0.16	6.423	2888.00	0.000	0.99
	No	2807	3.32	1.38	0.03				
Car	Yes	330	3.73	1.35	0.07	5.318	2888.00	0.000	0.43
	No	2560	3.30	1.39	0.03				
Children	Yes	356	3.78	1.41	0.07	6.254	2888.00	0.000	0.49
	No	2534	3.29	1.38	0.03				
Construction	Yes	270	4.02	1.57	0.10	7.528	311.48	0.000	0.75
	No	2620	3.28	1.35	0.03				
Dog bark	Yes	33	3.34	1.35	0.24	−0.016	2888.00	0.987 ^1^	0.00
	No	2857	3.35	1.39	0.03				
Footsteps	Yes	1118	3.18	1.26	0.04	−5.221	2620.68	0.000	−0.27
	No	1772	3.45	1.46	0.03				
General traffic	Yes	1166	3.63	1.34	0.04	9.037	2888.00	0.000	0.47
	No	1724	3.16	1.40	0.03				
Horn	Yes	76	3.94	1.36	0.16	3.802	2888.00	0.000	0.61
	No	2814	3.33	1.39	0.03				
Laughter	Yes	358	3.56	1.37	0.07	3.109	2888.00	0.002	0.24
	No	2532	3.32	1.39	0.03				
Motorcycle	Yes	169	4.17	1.41	0.11	8.028	2888.00	0.000	0.88
	No	2721	3.30	1.38	0.03				
Music	Yes	222	4.01	1.33	0.09	7.437	2888.00	0.000	0.72
	No	2668	3.29	1.38	0.03				
Non-identifiable	Yes	251	3.11	1.43	0.09	−2.818	2888.00	0.005	−0.26
	No	2639	3.37	1.39	0.03				
Other	Yes	391	3.21	1.29	0.07	−2.256	544.31	0.024	−0.16
	No	2499	3.37	1.41	0.03				
Rail	Yes	77	3.70	1.26	0.14	2.237	2888.00	0.025	0.36
	No	2813	3.34	1.39	0.03				
Rustling leaves	Yes	106	2.85	1.40	0.14	−3.749	2888.00	0.000	−0.52
	No	2784	3.37	1.39	0.03				
Screeching brakes	Yes	52	3.98	1.32	0.18	3.295	2888.00	0.001	0.64
	No	2838	3.34	1.39	0.03				
Shouting	Yes	199	4.21	1.45	0.10	9.139	2888.00	0.000	0.92
	No	2691	3.28	1.37	0.03				
Siren	Yes	95	3.72	1.38	0.14	2.661	2888.00	0.008	0.39
	No	2795	3.33	1.39	0.03				
Speech	Yes	1949	3.43	1.35	0.03	4.431	1741.72	0.000	0.25
	No	941	3.18	1.46	0.05				
Ventilation	Yes	146	3.32	1.31	0.11	−0.265	2888.00	0.791 ^1^	−0.03
	No	2744	3.35	1.40	0.03				
Water	Yes	344	3.51	1.53	0.08	2.190	421.05	0.029	0.19
	No	2546	3.32	1.37	0.03				

^1^ Differences for these sound sources are not statistically significant (*p* > 0.05); all other differences are.

## Data Availability

The data presented in this study are openly available at this link: https://zenodo.org/record/7158057 (accessed on: 7 October 2022).
